# Magnetic Vortices as Efficient Nano Heaters in Magnetic Nanoparticle Hyperthermia

**DOI:** 10.1038/s41598-017-18162-8

**Published:** 2018-01-19

**Authors:** N. A. Usov, M. S. Nesmeyanov, V. P. Tarasov

**Affiliations:** 1 National University of Science and Technology «MISiS», 119049 Moscow, Russia; 2 Pushkov Institute of Terrestrial Magnetism, Ionosphere and Radio Wave Propagation, Russian Academy of Sciences, IZMIRAN, 142190, Troitsk, Moscow, Russia; 3 National Research Nuclear University “MEPhI”, 115409 Moscow, Russia

## Abstract

Magnetic vortices existing in soft magnetic nanoparticles with sizes larger than the single-domain diameter can be efficient nano-heaters in biomedical applications. Using micromagnetic numerical simulation we prove that in the optimal range of particle diameters the magnetization reversal of the vortices in spherical iron and magnetite nanoparticles is possible for moderate amplitudes of external alternating magnetic field, *H*_0_ < 100 Oe. In contrast to the case of superparamagnetic nanoparticles, for the vortex configuration the hysteresis loop area increases as a function of frequency. Therefore, high values of the specific absorption rate, on the order of 1000 W/g, can be obtained at frequencies *f* = 0.5–1.0 MHz. Because the diameter *D* of a non single-domain particle is several times larger than the diameter *d* of a superparamagnetic particle, the volume of heat generation for the vortex turns out to be (*D*/*d*)^3^ times larger. This shows the advantage of vortex configurations for heat generation in alternating magnetic field in biomedical applications.

## Introduction

Magnetic nanoparticles are promising for theranostic applications and image-guided therapy as they combine imaging capability with therapeutic properties^1–6^. Due to their small sizes magnetic nanoparticles can accumulate in many tumor tissues by means of a passive mechanism known as the enhanced permeability and retention effect^4^. The application of external magnetic field gradient enables one to manipulate the nanoparticle position in biological media as the nanoparticle experiences a magnetic force resulting in magnetophoretic mobility. Besides, magnetic nanoparticles can generate large amount of thermal energy under the influence of alternating magnetic field of optimal frequency and amplitude^5,6^. The local heating of biological tissues suppresses the growth of tumors and destroys them completely. Due to these remarkable properties magnetic nanoparticles can combine several theranostic functionalities such as magnetic resonance imaging contrast enhancement, targeted drug delivery and magnetic hyperthermia.

The local increase of tissue temperature might be achieved using ultrasound, microwaves, or near-infrared radiation^7^. Recently, new biodegradable 2D nanomaterials such as black phosphorus quantum dots^8,9^ and antimonene quantum dots^10^ with high photothermal conversion efficiency were suggested to use as photothermal agents in cancer photothermal therapy. However, magnetic hyperthermia has important advantages^1–4,6^ over other methods of cancer treatment. First of all, the penetration depth of low frequency alternating magnetic field is much higher than that for light, infrared radiation or acoustic waves. Therefore much deeper tissues can be heated locally. Next, the specific absorption rate (SAR) of optimized assemblies of biodegradable magnetic nanoparticles can reach very high values, of the order of 1000 W/g^4–6^. Thus, positive therapeutic effect can be achieved with relatively small amount of magnetic nanoparticle reducing potential toxicity of nanomaterials *in vivo*. Finally, magnetic nanoparticles can be used to generate local heat resulting in the release of drugs either bound to the magnetic nanoparticle or encapsulated within polymeric matrices^11^.

In magnetic hyperthermia it is desirable to get useful therapeutic effect with the lowest possible magnetic nanoparticle concentration in biological media. Therefore, it is preferable to use magnetic nanoparticle with sufficiently large SAR value. It is important also to ensure the magnetization reversal of magnetic nanoparticle assembly in an alternating magnetic field of moderate amplitude, *H*_0_ < 100–200 Oe. Indeed, the use of strong alternating magnetic field requires generation of sufficiently large electric currents. It might be dangerous in a clinic. In addition, according to the empirical Brezovich’s criterion^6,12^, the alternating magnetic field is harmless to the human body if its amplitude and frequency *f* satisfy the condition *fH*_0_ < 5×10^9^ A/(ms). Therefore, it is the superparamagnetic nanoparticles with diameters substantially smaller than the single-domain diameter *D*_*c*_ that have been investigated in recent experimental and theoretical studies in magnetic hyperthermia^13–26^. Actually, the coercive force of superparamagnetic nanoparticles with diameters *d* < *D*_*c*_ is known to decrease significantly at a room temperature under the influence of thermal fluctuations of their magnetic moments.

In this paper, we draw attention to a possibility of using non single-domain nanoparticles with diameters *D* > *D*_*c*_ for local heating of the biological media. Vortex configuration has the lowest total energy for nanoparticles of soft magnetic type with diameters *D* > *D*_*c*_,^27–33^. Though the average magnetization of the vortex rapidly decreases with increasing particle diameter, it remains appreciable, < *M* > /*M*_*s*_ > 0.3–0.5, for particles with diameters close to the single-domain one.

At present, the nanoparticles of iron and iron oxides are considered to be most promising for use in magnetic hyperthermia because of their low toxicity^4,6,34^. Therefore, we study theoretically the behavior of vortices in spherical iron and magnetite nanoparticles in alternating magnetic field. In the case of magnetite, the nanoparticles of cubic shape are also considered. In the experiment sufficiently large cubic nanoparticles can be obtained by various methods^17,20,22,35–37^. Apparently, these particles have a perfect crystal structure^36^ as their magnetic characteristics are close to the corresponding values for bulk material^38^.

It has been found recently^6,39–41^ that being embedded in a biological environment, for example, into a tumor, magnetic nanoparticles turn out to be tightly bound to the surrounding tissues. Therefore, the rotation of magnetic nanoparticles as a whole under the influence of alternating external magnetic field is greatly suppressed. In such a case, the Brownian relaxation is unimportant^6^, and only the evolution of the particle magnetization under the influence of an alternating magnetic field has to be considered. In this paper the numerical simulations of vortex configurations and low frequency hysteresis loops are carried out based on the solution of the Landau-Lifshitz-Gilbert (LLG) equation^31,42^. It is shown that in an optimal range of particle diameters the magnetization reversal of the vortex is possible for moderate amplitudes of external alternating magnetic field, *H*_0_ < 100–200 Oe. In contrast to the case of superparamagnetic nanoparticles^43^, the area of the low frequency hysteresis loops for vortex increases with increase of alternating field frequency. As a result, very large SAR values, of the order of 1000 W/g, have been obtained at frequencies *f* ~ 0.5–1.0 MHz. It is worth mentioning also that because the diameter *D* of a non single-domain particle is several times larger than the diameter *d* of a superparamagnetic particle, the volume of heat generation turns out to be (*D*/*d*)^3^ times larger for vortex configuration.

These results seem important for further successful development of magnetic nanoparticle hyperthermia.

## Results and Discussion

### Spherical iron nanoparticles

**Figure 1:**
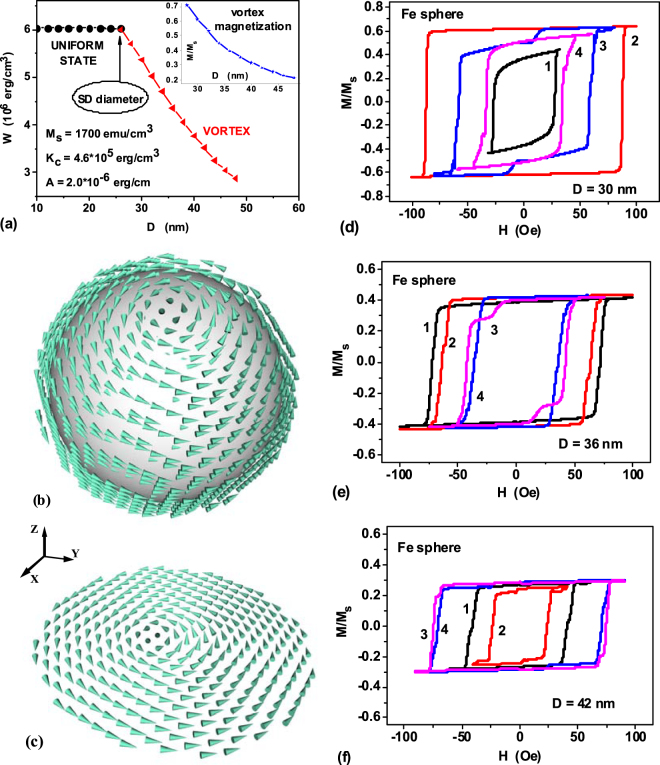
(**a**) Energy diagram of equilibrium magnetization states in a spherical iron nanoparticle with cubic anisotropy. The insert shows the reduced average magnetic moment of the vortex as a function of the nanoparticle diameter. (**b**) General view of the vortex in iron nanoparticle of diameter *D* = 42 nm; (**c**) The diametrical cross section of the vortex perpendicular to the easy anisotropy axis. (**d–f**) Hysteresis loops of vortex in iron nanoparticles of different diameters for some characteristic directions of applied magnetic field: 1) *ω*_*h*_ = *ψ*_*h*_ = 0.0; 2) *ω*_*h*_ = 0.955, *ψ*_*h*_ = *π*/4; 3) *ω*_*h*_ = *π*/4, *ψ*_*h*_ = 0.0; 4) *ω*_*h*_ = *ψ*_*h*_ = *π*/8. The frequency and amplitude of alternating magnetic field are given by *f* = 1 MHz and *H*_0_ = 100 Oe, respectively.

Let us first consider iron nanoparticles, which are especially interesting for use in magnetic hyperthermia^14–16^ due to the high saturation magnetization of iron, *M*_*s*_ = 1700 emu/cm^3^. The cubic magnetic anisotropy constant of iron is assumed to be^38^ *K*_*c*_ = 4.6×10^5^ erg/cm^3^, the exchange constant is *A* = *С*/2 = 2.0×10^−6^ erg/cm. Figure 1a shows the energy diagram of stable micromagnetic states existing in a spherical iron nanoparticle with cubic anisotropy, depending on its diameter. According to Fig. 1a, for spherical iron nanoparticles the single-domain diameter approximately equals *D*_*c*_ ≈ 26 nm. This value, determined numerically, is in agreement with the analytical estimates^44^. The insert in Fig. 1a shows that in the range of diameters *D* = 30–40 nm the average reduced magnetization of the vortex, although decreasing as a function of the particle diameter, remains sufficiently large, < *M* > /*M*_*s*_ > 0.3.

The magnetization distribution for the vortex in spherical iron nanoparticle of diameter *D* = 42 nm, obtained by means of numerical simulation, is shown in Fig. 1b,c. In the cylindrical core of the vortex the particle magnetization remains approximately homogeneous. This region gives the main contribution to the remanent vortex magnetization. The outer shell of the vortex where the magnetization has an azimuthal direction does not contribute to the remanent magnetization of the particle.

It is well known^42^, that for a uniformly magnetized iron nanoparticle with positive cubic anisotropy constant, *K*_*c*_* > *0, the easy anisotropy axes are parallel to the Cartesian coordinate axes, i.e. ( ± 1,0,0), etc. It is interesting to note that in a small range of diameters, *D*_*c*_ < *D* < 32 nm, the vortex axis is oriented along one of the cube diagonals. However, as the diameter of the nanoparticle increases, *D* > 32 nm, the total energy of the vortex decreases if its axis is directed along one of the easy axes of cubic anisotropy.

To calculate the low-frequency hysteresis loops of iron nanoparticles with diameters *D* > *D*_*c*_ it is necessary to study the vortex dynamics in alternating magnetic field. Note that for particles with cubic anisotropy the region of nonequivalent directions of the external magnetic field with respect to the orientations of the easy anisotropy axes in the spherical coordinates (*ψ*_*h*_, *ω*_*h*_) is bounded^31^ by the spherical triangle Ω: 0 ≤ *ψ*_*h*_ ≤ *π*/4; 0 ≤ *ω*_*h*_ ≤ arctan(1/cos(*ψ*_*h*_)). Therefore, it is sufficient to study the low-frequency hysteresis loops of vortices for directions of the external magnetic field within spherical triangle Ω.

Figure 1d–f show calculated hysteresis loops of iron nanoparticles of different diameters, *D* = 30, 36 and 42 nm, respectively. The loops in Fig. 1d–f are calculated for some characteristic directions of the magnetic field lying in the corners and inside the spherical triangle Ω. One can see that in all particular cases considered the particle coercive force at the frequency *f* = 1 MHz does not exceed 100 Oe, which is appealing for application in magnetic hyperthermia. As Fig. 1d–f show, with increasing nanoparticle diameter, the hysteresis loop area gradually decreases, as the average magnetization of the vortex decreases as a function of particle diameter. Nevertheless, it remains large enough even for nanoparticles of diameter *D* = 42 nm. As shown below, in the range of diameters *D* = 30–42 nm, the calculated SAR of a dilute random assembly of iron nanoparticles is of the order of 1000 W/g at frequencies *f* > 0.5–1 MHz.

### Spherical magnetite nanoparticles

**Figure 2:**
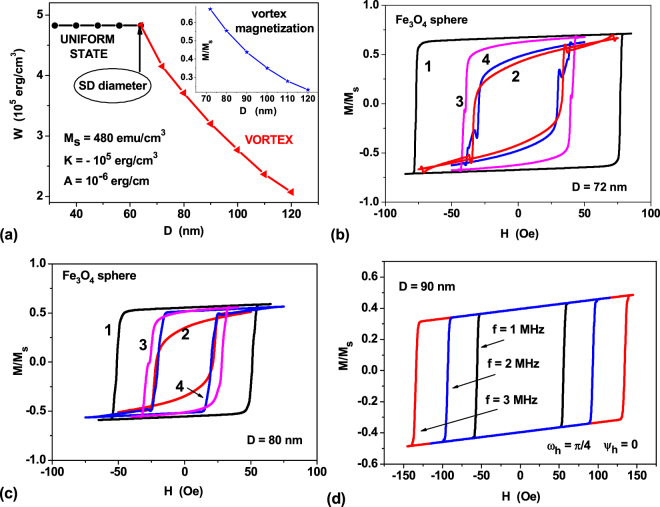
(**a**) Energy diagram of equilibrium magnetization states in a spherical magnetite nanoparticle. The insert shows the average reduced magnetic moment of the vortex in magnetite nanoparticle depending on the particle diameter. (**b**) The vortex hysteresis loops for spherical magnetite nanoparticles with diameters *D* = 72 nm for some characteristic directions of the external magnetic field: 1) *ω*_*h*_ = *ψ*_*h*_ = 0.0; 2) *ω*_*h*_ = 0.955, *ψ*_*h*_ = *π*/4; 3) *ω*_*h*_ = *π*/4, *ψ*_*h*_ = 0.0; 4) *ω*_*h*_ = *ψ*_*h*_ = *π*/8. (**c**) the same as in b) but for particle diameter *D* = 80 nm. (**d**) The hysteresis loops of magnetite nanoparticle with diameter *D* = 90 nm for various frequencies at *ω*_*h*_ = π/4, *ψ*_*h*_ = 0.0.

Similar results are obtained for spherical nanoparticles of magnetite, Fe_3_O_4_, which are considered^1–6^ to be the most suitable for biomedical applications. The saturation magnetization of magnetite nanoparticles equals to the bulk value^38^, *M*_*s*_ = 480 emu/cm^3^, the cubic magnetic anisotropy constant is negative, *K*_*c*_ = −1.0×10^5^ erg/cm^3^, the exchange constant is *A* = 1.0×10^−6^ erg/cm. For particles with negative cubic anisotropy constant the easy anisotropy axes are parallel to the cube diagonals. As Fig. 2a shows, in agreement with analytical estimates^44^ the single-domain diameter of magnetite nanoparticle, *D*_*c*_ ≈ 64 nm, is much higher as compared to that of iron nanoparticle because of significantly lower value of the saturation magnetization of magnetite. As inset in Fig. 2a shows, in the range of diameters *D* = 70–100 nm, the average magnetization of magnetite nanoparticles remains appreciable. This should result in low-frequency hysteresis loops of a sufficiently large area. As an example of calculations performed, Fig. 2b and c show hysteresis loops of magnetite nanoparticles with diameters *D* = 72 nm and 80 nm at frequency *f = *1 MHz and *H*_0_ = 100 Oe for some characteristic directions of the alternating magnetic field in the spherical triangle Ω. One can see in Fig. 2,c that the coercive force of the calculated hysteresis loops does not exceed 100 Oe. Similar results were obtained for magnetite nanoparticles in the range of diameters *D* = 70–100 nm.

It is interesting to note that, in contrast to superparamagnetic nanoparticles, for which the area of the hysteresis loop at moderate values of the alternating field amplitude, *H*_0_ ≤ 100 Oe, falls^43^ with increasing frequency in the interval *f* > 0.5–1 MHz, the vortex hysteresis loop area grows as a function of the frequency. As an example, Fig. 2d shows the behavior of the vortex hysteresis loops at various frequencies for magnetite nanoparticle with diameter *D* = 90 nm for one of the characteristic directions of the alternating magnetic field in the spherical triangle Ω. Similar results were also obtained for particles of different diameters and different directions of the alternating magnetic field. Therefore, for vortex it is reasonable to increase the frequency of the alternating magnetic field, while decreasing simultaneously the field amplitude. Note that decreasing of the alternating field amplitude is preferable for technical reasons, since this reduces the cost and increases the safety of the equipment to be used in medical practice.

### Magnetite nanocubes

Modern methods of chemical synthesis of magnetic nanoparticles allow the growth of magnetite nanoparticles of cubic shape^17,20,22,35,36^ with the size *L* from 18 to 160 nm. Moreover, these magnetite nanoparticles are of perfect quality^36^ as their saturation magnetization is close to the corresponding bulk value^38^. Therefore, we carried out also the calculation of the equilibrium magnetization distributions and low frequency hysteresis loops for magnetite nanoparticles of cubic shape.

**Figure 3:**
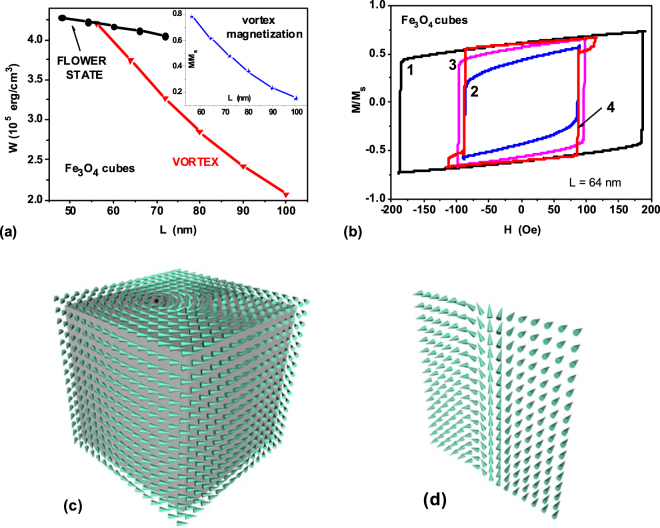
(**a**) Energy diagram of the equilibrium magnetization states in a cubic magnetite nanoparticle. The insert shows the average reduced magnetic moment of the vortex depending on the length of the cube’s side. (**b**) Hysteresis loops of vortex configuration in a cubic magnetite nanoparticle with size *L* = 64 nm for some characteristic directions of external magnetic field: 1) *ω*_*h*_ = *ψ*_*h*_ = 0.0; 2) *ω*_*h*_ = 0.955, *ψ*_*h*_ = *π*/4; 3) *ω*_*h*_ = *π*/4, *ψ*_*h*_ = 0.0; 4) *ω*_*h*_ = *ψ*_*h*_ = *π*/8. (**c**) the vortex magnetization distribution in a cubic magnetite nanoparticle with size *L* = 64 nm. (**d**) the cross-section of the vortex shown in c) along the vortex core.

As Fig. 3a shows, quasi-uniform flower state^45,46^ exists in sufficiently small cubic nanoparticles of soft magnetic type. At larger sizes, the vortex competes in energy with the flower state. The intersection of the curves corresponding to the flower state and vortex determines the effective single-domain size^31^ for cubic-shaped nanoparticles. According to Fig. 3a, for cubic magnetite nanoparticle the effective single-domain size is *L*_*c*,*ef*_ = 56 nm. It is smaller than the single-domain diameter of spherical magnetite nanoparticle, *D*_*c*_ = 64 nm.

Figure 3b shows the hysteresis loops of a cubic magnetite nanoparticle with the size *L* = 64 nm for some characteristic directions of an external magnetic field in a spherical triangle Ω. The frequency and amplitude of the alternating magnetic field are given by *f* = 1 MHz and *H*_0_ = 200 Oe, respectively. Figure 3d show an example of the vortex magnetization distribution in cubic magnetite nanoparticle in the ground state, in the absence of an external magnetic field. In the case of cubic magnetite nanoparticles the coercive force of low frequency hysteresis loops for certain directions of the external magnetic field turns out to be larger than that for the case of spherical nanoparticles. To obtain complete hysteresis loops it was necessary to increase the amplitude of the alternating magnetic field up to *H*_0_ = 200 Oe.

**Figure 4:**
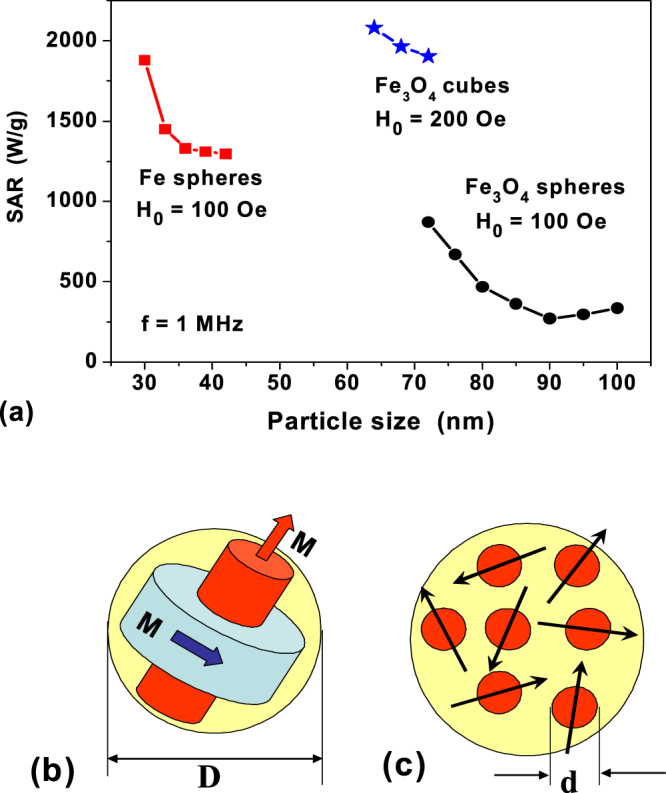
(**a**) SAR of dilute randomly oriented assemblies of iron and magnetite nanoparticles in vortex states as a function of the particle diameter. (**b**) The heat generation volume for a vortex in a nanoparticle of diameter *D* > *D*_*c*_, (**c**) A cluster of the same diameter *D* consisting of superparamagnetic nanoparticles with significantly smaller diameters *d* ≪ *D*_*c*_.

Comparing the magnetic properties of spherical magnetite nanoparticles and magnetite nanocubes one notes in Figs 2a and 3a that the single domain diameter for spherical magnetite nanoparticles equals *D*_*c*_ = 64 nm, whereas for magnetite nanocubes the effective single domain size decreases up to *L*_*c*,*ef*_ = 56 nm. It is even more important that as Figs 2 and 3 show the shape and area of the low frequency vortex hysteresis loops for these nanoparticles differ considerably due to their different external shape. As Fig. 4a shows, for application in magnetic hyperthermia the optimal particle sizes for magnetite nanocubes correspond to the range of 65 nm < *L* < 75 nm. For spherical magnetite nanoparticles the optimal particle diameters are found to be higher, 75 nm < *D* < 100 nm.

### Specific absorption rate

The area *A* of a particular hysteresis loop can be calculated in the variables (*M*/*M*_*s*_, *H*). The corresponding SAR is then calculated^6,43^ as $SAR={10}^{-7}{M}_{s}fA/\rho$ (W/g), where *ρ* is the nanoparticle density. It is given by *ρ* = 7.88 g/cm^3^ for iron nanoparticles and *ρ* = 5.0 g/cm^3^ for magnetite nanoparticles, respectively. Having calculated a sufficient number of the low frequency hysteresis loops with the direction of the magnetic field within the spherical triangle Ω, it is possible to calculate the SAR of a dilute randomly oriented assembly of magnetic nanoparticles being in the vortex states. Figure 4a shows the dependence of SAR of dilute randomly oriented assemblies of spherical iron and magnetite nanoparticles, as well as cubic magnetite nanoparticles as a function of particle size. The SAR averaging was carried out over 10–12 independent magnetic field directions within the spherical triangle Ω. As Fig. 4a shows, for a dilute assembly of iron nanoparticles in the range of diameters *D* = 30–42 nm the SAR exceeds the value of 1000 W/g at a frequency *f* = 1 MHz. For magnetite nanoparticles, the SAR values also remain high, at least for nanoparticle diameters close to the single-domain one. Figure 4a also shows the SAR of dilute randomly oriented assembly of cubic magnetite nanoparticles as a function of the cube side. As can be seen from Fig. 4a, in the investigated range of sizes *L* = 64–72 nm the SAR of the assembly reaches values of the order of 2000 W/g at the frequency *f* = 1 MHz and amplitude *H*_0_ = 200 Oe.

Note that the use of non-single-domain magnetic nanoparticles can be advantageous in magnetic hyperthermia, because as Fig. 4b and c show the physical volume of heat generation for such nanoparticles is significantly greater than that for the corresponding superparamagnetic nanoparticles. For example, for slightly elongated superparamagnetic magnetite nanoparticles the maximum SAR at frequencies of the order of *f* = 0.5 MHz occurs for particles with diameter *d* = 13 nm^43^. On the other hand, the diameters *D* = 70–75 nm are optimal for vortex state in magnetite nanoparticles. Accordingly, the ratio of the volumes of these particles is (*D*/*d*)^3^ ≈ 160. As Fig. 4c shows, a large number of small superparamagnetic nanoparticles of diameter *d* can be distributed in the volume of a sphere of diameter *D*. However, both the experiment and calculations^6,16,47,48^ show that the SAR of dense clusters of magnetic nanoparticles significantly decreases under the influence of magneto- dipole interaction.

It is interesting to note that the heating ability in alternating magnetic field of rather big nanoparticles of iron and iron oxide was recently studied experimentally. In ref.^18^ iron and magnetite nanoparticles were prepared by physical solar vapor deposition method. The mean diameter of iron nanoparticles was tuned between 45 and 85 nm. According to Fig. 1a these sizes exceed considerably the single domain diameter of iron nanoparticles, *D*_*c*_ = 26 nm. Therefore, these nanoparticles have to be in vortex magnetization state. Experimentally rather small SAR values, of the order of 70 W/g, were measured in iron nanoparticles with diameter *D* = 50 nm at a frequency *f* = 765 kHz and magnetic field amplitude *H*_0_ = 300 Oe. This result is in accordance with the present calculations, because for non single domain iron nanoparticles the optimal diameters were found to be in the range *D* = 30–35 nm. On the other hand, the average diameter of magnetite nanoparticles was around 75 nm, i.e. also higher than the corresponding single domain diameter, *D*_*c*_ = 64 nm (see Fig. 2a). However, the diameter *D* = 75 is close to the optimal diameters for non single domain magnetite nanoparticles, *D* = 75–100 nm. As a result, at the same frequency and magnetic field amplitude the SAR of magnetite nanoparticles with average diameter *D* = 75 nm was found to be around 900 W/g.

In another study^20^ magnetite nanocubes with a mean size of 79 and 124 nm were synthesized and transferred to water by silica surface modification. According to Fig. 3a, these nanoparticles have to be in vortex magnetization states. High SAR values ranging from 560 to 1160 W/g were obtained for these nanoparticles in water suspensions at a frequency *f* = 360 kHz and magnetic field amplitude *H*_0_ = 560 Oe.

## Conclusions

Nanotechnology has a growing impact on medicine in general and on the diagnosis and treatment of cancer in particular^1–6^. In magnetic hyperthermia the optimized assemblies of magnetic nanoparticles are used for remote local generation of heat to kill malignant cells keeping the surrounding tissue healthy. Two regimes of thermal treatment have been investigated in preclinical experiments, i.e. thermal ablation and hyperthermia^4^. The application of high temperatures, above 50 − 60 °C, corresponds to the thermal ablation regime, which results in coagulation, protein denaturation, and necrosis of the malignant tissue^2,4^. In magnetic hyperthermia the temperature rise typically reach 42–43 °C. The inactivation of protein synthesis, inhibition of DNA repair processes, and alteration of membrane permeability are among the mechanisms responsible for cell death caused by magnetic hyperthermia^49^. It was shown also^50^ that hyperthermia treatment can induce tumor-specific immune responses as a result of heat-shock protein expression. In the experimental rat glioma model the T-9 cells were transplanted into each femur of rats used in the experiments. Although only one tumor was subjected to hyperthermia, the other tumor also disappeared completely. These results suggest that hyperthermia can kill not only local tumors exposed to heat treatment, but also tumors at distant sites, including metastatic cancer cells.

At present, the use of iron oxide nanoparticles for magnetic hyperthermia has been authorized in the European Union to treat glioblastoma^51^ and prostate cancer^52^. In the clinic of Dr. A. Jordan in Berlin, Germany, a special applicator for humans has been developed to treat prostate cancer. It operates at frequency *f* = 100 kHz and amplitude of alternating magnetic field *H*_0_ = 100–200 Oe. Promising therapeutic results have been obtained also when magnetic hyperthermia is combined with radiotherapy or chemotherapy^4,53^.

It has been proved^4,34^ that biodegradation processes related with the exposure of nanoparticles to acid environments in living organisms lead to a deterioration of their magnetic properties. The structural degradation of individual magnetic nanoparticles is driven by a stochastic corrosion process at the nanoparticle surfaces. As a result, the magnetic properties of biodegraded magnetic nanoparticles can be significantly altered with time under acidic conditions^34^. Nevertheless, depending on the nature and thickness of particle coating, the time window where the intrinsic magnetic characteristics of nanoparticles are preserved can be made as long as several days^4,6^ before the chemical changes decrease considerably their heating capabilities.

However, in practice it is not easy to obtain a sufficient amount of heating power in biological media in particular due to the restrictions for the application of very high frequencies and strong magnetic fields, in order to avoid nonspecific heating of healthy tissues^1,4–6,12^. It is also important to get useful therapeutic effect with the lowest possible magnetic nanoparticle concentration in biological media. The optimization of the heating ability of magnetic nanoparticle assemblies has been recently achieved through novel design and composition of the nanomaterials^6,17,21–26^. In this paper, we draw attention to a possibility of using relatively big, non single-domain nanoparticles, being in vortex magnetic configurations, for effective local heating of the biological media in magnetic hyperthermia.

The vortex magnetization distributions in magnetically soft nanoparticles have long been studied both theoretically and experimentally^27–33^. These states are of fundamental importance, as the vortices have the lowest total energy in these nanoparticles of sufficiently large diameters. However, up to now, vortices in magnetically soft nanoparticles have not found practical application. It seems therefore interesting if the vortices can be used to generate heat in an alternating magnetic field in magnetic hyperthermia.

In this paper the low-frequency hysteresis loops of vortices existing in iron and magnetite nanoparticles have been investigated by means of numerical simulation. It is shown that for a dilute assemblies of these nanoparticles in the optimal range of diameters it is possible to obtain very high SAR values, on the order of 1000 W/g at frequencies *f* ~ 0.5–1.0 MHz and moderate alternating magnetic field amplitudes, *H*_0_ < 100 Oe. As we mentioned above for technical and medical requirements it is desirable to reduce the amplitude of the alternating magnetic field acting on the nanoparticles. To maintain sufficiently high SAR values, the decrease in the alternating field amplitude can be compensated by increasing field frequency. In this connection, the use of vortices in magnetic hyperthermia may be preferable, since with increasing frequency the hysteresis loop area for vortices increases, in contrast to the assembly of superparamagnetic nanoparticles. Indeed, for an assembly of superparamagnetic nanoparticles the hysteresis loop area decreases^43^ with increasing frequency in the interval *f* > 0.5–1.0 MHz for magnetic field amplitudes *H*_0_ < 100 Oe.

It is also important to note that in the case of non single-domain nanoparticles with diameters *D* > *D*_*c*_ the volume of the heat generation is (*D*/*d*)^3^ times larger than that for small superparamagnetic nanoparticle of diameter *d* < *D*_*c*_. In a dense assembly of superparamagnetic nanoparticles a significant decrease of the SAR occurs^41,47,48^ due to the influence of mutual magneto- dipole interactions of the nanoparticles. At the same time, one can expect that the effect of the magnetostatic interactions will not be so significant for the vortex states, since the magnetic moments of the vortices are relatively small.

## Methods

Dynamics of the unit magnetization vector $\vec{\alpha }(\vec{r})$ of a non single domain nanoparticle in applied magnetic field ${\vec{H}}_{0}\,\sin (\omega t)$ is described by the LLG equation^31,42^

1 $$\frac{\partial \vec{\alpha }}{\partial t}=-\gamma (\vec{\alpha }\times {\vec{H}}_{ef})+\kappa (\vec{\alpha }\times \frac{\partial \vec{\alpha }}{\partial t}),$$

where *γ* is the gyromagnetic ratio and *κ* is the phenomenological damping constant. The effective magnetic field ${\vec{H}}_{ef}$ acting on the unit magnetization vector can be calculated as a derivative of the total nanoparticle energy^42^

2 $${\vec{H}}_{ef}=-\frac{\partial W}{V{M}_{s}\partial \vec{\alpha }},\quad {M}_{s}{\vec{H}}_{ef}=C{\rm{\Delta }}\vec{\alpha }-\frac{\partial {w}_{a}}{\partial \vec{\alpha }}+{M}_{s}({\vec{H}}_{0}\,\sin (\omega t)+\vec{H}^{\prime} )$$

Here *V* is the nanoparticle volume, *M*_*s*_ is the saturation magnetization, *C* is the exchange constant, and $\vec{H}^{\prime}$ is the demagnetizing field. The magneto-crystalline anisotropy energy density of a nanoparticle with cubic anisotropy is given by^42^

3 $${w}_{a}={K}_{c}({\alpha }_{x}^{2}{\alpha }_{y}^{2}+{\alpha }_{x}^{2}{\alpha }_{z}^{2}+{\alpha }_{y}^{2}{\alpha }_{z}^{2}),$$

where *K*_*c*_ is the cubic anisotropy constant.

For numerical simulation a non single-domain nanoparticle is approximated by a set of small ferromagnetic cubes of side *b* much smaller than the exchange length ${L}_{ex}=\sqrt{C}/{M}_{s}$ of the ferromagnetic material. Typically, several thousands of numerical cells, *N* ~ 10^3^–104, is necessary to approximate with sufficient accuracy the vortex type magnetization distribution in nanoparticle volume. For reliable calculation of the low frequency hysteresis loops of the nanoparticle it is important to keep the numerical time step Δ*t* sufficiently small^39^ with respect to the characteristic precession time of the unit magnetization vectors in various numerical cells, *T*_*p*_ ~ 1/*γ* < *H*_*ef*_ > , where < *H*_*ef*_ > is the average value of the effective magnetic field of the nanoparticle. In the present calculations the numerical time step is fixed at Δ*t*/*T*_*p*_ = 1/30, the magnetic damping parameter being *κ* = 0.5.

The equilibrium micromagnetic configurations for the nanoparticles studied were calculated using the same LLG equation with zero applied magnetic field. In accordance with the Eq. (1), the final magnetization state is assumed to be stable under the condition

4 $${\max }_{(1\le i\le N)}|[{\vec{\alpha }}_{i}\times {\vec{H}}_{ef,i}/\Vert {\vec{H}}_{ef,i}\Vert ]| < {10}^{-6},$$

where ${\vec{\alpha }}_{i}$ and ${\vec{H}}_{ef,i}$ are the unit magnetization vector and effective magnetic field in the *i*-th numerical cell, respectively.

### Data availability statement

No datasets were generated or analysed during the current study.
